# Intoxication with 3-MeO-PCP alone: A case report and literature review

**DOI:** 10.1097/MD.0000000000018295

**Published:** 2019-12-27

**Authors:** Antoine Berar, Jean-Sébastien Allain, Sophie Allard, Charles Lefevre, Alain Baert, Isabelle Morel, Renaud Bouvet, Thomas Gicquel

**Affiliations:** a CHU Rennes, Department of Forensic Medicine; b CHU Rennes, Department of Internal Medicine and Clinical Immunology; c CIC-P 1414 Clinical Investigation Center, Inserm; d CHU Rennes, Department of Forensic Toxicology; e Université de Rennes, INRA, Inserm, Institut NuMeCan – UMR_A 1341, UMR_S 1241; f Université de Rennes, IDPSP – EA 4640, Rennes, France.

**Keywords:** 3-MeO-PCP, drug confusion, new psychoactive substances (NPS), research chemicals

## Abstract

**Rationale:**

3-Methoxyphencyclidine (3-MeO-PCP) is a new psychoactive substance derived from phencyclidine. Although it can lead to severe intoxications, the main manifestations and optimal management have not been well characterized. Here, we report 2 cases of 3-MeO-PCP intoxication in the same patient, and summarize the manifestations of this intoxication reported in literature.

**Patient concerns:**

A 17-year-old male purchased a bag of 3-MeO-PCP on the Internet but took an oral dose (200 mg) that corresponds to the less active isomer 4-MeO-PCP. He developed high blood pressure (158/131 mm Hg), tachycardia (100 bpm), and neurological manifestations (confusion, hypertonia, nystagmus, and then agitation). A maculopapular rash appeared, although this may have been related to the administration of midazolam. Hyperlactatemia (2.6 mmol/L) was the main laboratory finding. Seven days later, he returned to the emergency department after sniffing 50 mg of 3-MeO-PCP. High blood pressure, tachycardia, and neurological manifestations (psychomotor impairment and dysarthria) were present but less severe than after the first intoxication.

**Diagnosis:**

In the first intoxication, the blood and urine 3-MeO-PCP concentrations were, respectively, 71.1 ng/mL and 706.9 ng/mL. Conventional toxicity tests were all negative. In the second intoxication, biological samples were not available.

**Interventions:**

In the first intoxication, treatment consisted of intravenous hydration and midazolam. The patient was transferred to an intensive care unit for monitoring. After the second intoxication, he was monitored for 12 hours.

**Outcomes:**

The patient's condition improved quickly in both cases.

**Lessons:**

These cases provide additional information on the manifestations of 3-MeO-PCP intoxication. These manifestations are mainly cardiovascular (high blood pressure, tachycardia) and neurological. The fact that second (50 mg) intoxication was less severe than the first (200 mg) is suggestive of a dose–effect relationship for 3-MeO-PCP. The first case also emphasizes the risk of dosing errors caused by the similarity between the names “3-MeO-PCP” and “4-MeO-PCP.”

## 1 Introduction

Intoxications by new psychoactive substances (NPSs, ie, drugs designed to mimic established illicit drugs) constitute a public health problem. These substances are not controlled under the international drug control conventions^[1]^ and thus, depending on the country, are not always prohibited. Many NPSs can be purchased easily and cheaply on the Internet. As of December 2018, a total of 888 NPSs had been reported to the United Nations Office on Drugs and Crime. These substances had been recorded in 119 countries and territories, on all continents.^[1]^

The NPSs’ main clinical and biochemical manifestations have not been extensively characterized, and the optimal management of intoxications is challenging. Indeed, victims of intoxication are not always willing or able to tell the medical team which substances they have consumed. Furthermore, NPSs are not identified by routine toxicological analyses. The fact that cases of confusion between NPSs have been reported shows that the consumers are not always aware of which substance(s) they have taken.^[2]^ Finally, multiple intoxication is frequent, making it difficult to identify the specific manifestations associated with specific compounds.[^3^,^4^,^5^,^6^]

The NPS 3-methoxyphencyclidine (3-MeO-PCP) is an arylcyclohexylamine analogue of phencyclidine (PCP).[^7^,^8^] It is a potent *N*-methyl-D-aspartate (NMDA) receptor antagonist,^[7]^ and is used recreationally as a dissociative hallucinogen.^[6]^ Nasal^[4]^ and oral[^5^,^9^,^10^] administration has been reported, as well as inhalation.^[11]^ Sublingual, intramuscular, and rectal administration routes have been described by drug users.^[12]^ The metabolic pathway of 3-MeO-PCP has also been described but there are no data on the levels or properties of metabolites.[^5^,^13^,^14^]

Several publications have reported on cases of 3-MeO-PCP intoxication, either alone or in combination with other substances.[^4^,^5^,^9^,^10^,^11^,^15^] Some of these intoxications were fatal.[^3^,^4^,^14^,^16^] However, there is a significant lack of accurate literature data on intoxications by this NPS.[^4^,^6^,^10^,^15^] Here, we describe the clinical and/or laboratory findings in 2 cases (in the same patient) of intoxication with 3-MeO-PCP. We also review of the literature concerning nonfatal intoxications with 3-MeO-PCP.

## 2 Case report

The patient was a 17-year-old French male with a history of illicit drug use (benzodiazepines, opiates, cannabis, and amphetamines). He was part of a group of young polydrug abusers, one of whom had died from a heroin overdose a few months earlier.

He was admitted to the emergency department following agitation and consciousness disorders. The patient alternated between hypotonic and hypertonic states, with mental fog, agitation, and tremor of the limbs. Next, he developed nystagmus and dysarthria, but not facial paralysis. Deep tendon reflexes were brisk. The pupils were symmetrical, reactive, and of intermediate size. There were no focal neurologic signs. The systolic/diastolic blood pressure was 158/131 mm Hg, the heart rate was 100 beats per minute, and the peripheral oxygen saturation (SpO_2_, as measured using pulse oximetry) was 99%. The patient had mottled skin on his knees. There was no fever, and no respiratory impairment. The electrocardiogram was normal.

A laboratory assessment showed hyperlactatemia (2.6 mmol/L) and elevated serum creatine kinase (CK, 290 IU/L; N: 20–200 IU/L). Blood gas analysis revealed respiratory acidosis (pH: 7.36; partial pressure of carbon dioxide [pCO_2_]: 43 mm Hg; bicarbonate: 25 mmol/L). There were no signs of liver or kidney failure (serum creatinine: 73 μmol/L; estimated glomerular filtration rate calculated according to the Chronic Kidney Disease Epidemiology Collaboration equation: 130 mL/minute/1.73 m^2^; prothrombin ratio: 99%; serum liver enzymes: normal). The complete blood count was normal.

Toxicological screening was negative. Urine and peripheral blood samples tested negative for alcohol, tricyclics, buprenorphine, barbiturates, amphetamines, methamphetamines, 3,4-methylenedioxymethamphetamine (MDMA, “ecstasy”), opiates, methadone, benzodiazepines, cocaine, cannabis, and paracetamol.

In view of the hypertonia, midazolam (1 mg) was administered by the emergency medical team. A total of 1500 mL of saline solution was infused. The patient was transferred to the intensive care unit. A maculopapular rash appeared on the chest 2 hours after midazolam had been administered. The serum CK level increased (to 339 IU/L), whereas the skin mottling disappeared and the lactatemia (1.60 mmol/L) and blood gas measurements (pH: 7.40; pCO_2_: 41 mm Hg; bicarbonate 27 mmol/L) normalized. The neutrophil count rose slightly, to 8.92 G/L (N: 1.8–8). Serum calcium and phosphorus levels were normal. Management of the agitation required the administration of sedative antipsychotics (cyamemazine and loxapine). Intravenous hydration therapy (3000 mL per 24 hours) was continued. In less than 24 hours, the maculopapular rash had disappeared, the neurological disorders regressed, and the blood pressure and heart rate normalized. The patient continued with mild sedation, which was withdrawn the following day.

**Figure 1:**
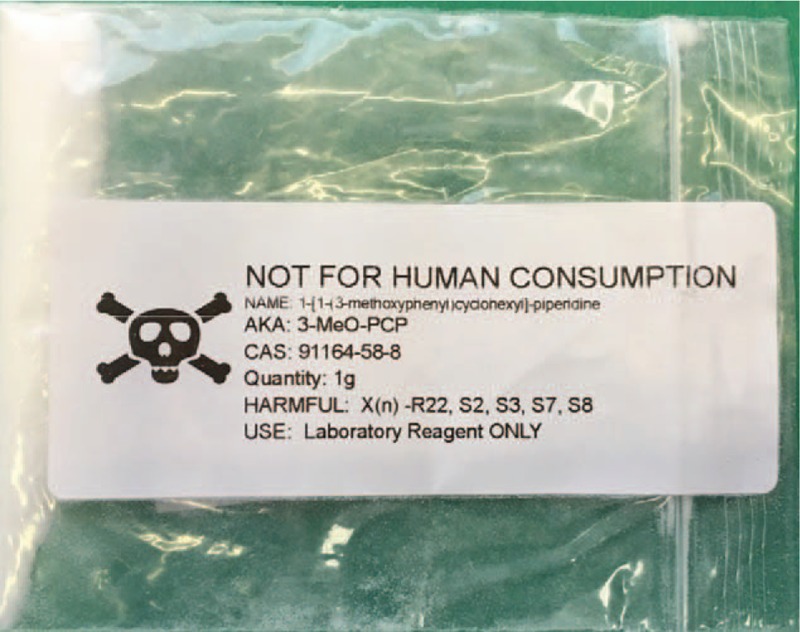
. The sachet labeled “3-MeO-PCP” found near to where the 17-year-old patient had been taken ill. 3-MeO-PCP = 3-methoxyphencyclidine.

The patient's father showed the medical team a 1-g sachet of the substance presumably consumed by the patient. The sachet bore a “poison” skull-and-crossbones symbol, and was labeled “3-MeO-PCP,” “not for human consumption,” and “Laboratory Reagent ONLY” (Fig. 1). The father had found it at home, near to where the patient had been taken ill. The sachet was sent to the toxicology laboratory for analysis. Thereafter, blood and urine tests confirmed the presence of this drug. A few hours after consumption, 3-MeO-PCP was quantified by liquid chromatography–high-resolution mass spectrometry (LC-HRMS), with 71.1 ng/mL in the peripheral blood and 706.9 ng/mL in the urine (corresponding to 539 μg/mg of creatinine).

When the patient had recovered, he told us that he had consumed 200 mg of 3-MeO-PCP, bought online from the Netherlands. This was surprising, because the usual dose of 3-MeO-PCP is between 2 and 15 mg.^[12]^ A subsequent search suggested that the usual dose of the isomer 4-MeO-PCP is between 75 and 250 mg, that is, similar to the dose of 200 mg taken by the patient.^[17]^ He referred by mistake to these doses instead of usual doses of 3-MeO-PCP.

The patient was first transferred to a psychiatric hospital and then discharged to home after a few days. One week later, he was again admitted to the emergency department after sniffing 50 mg of 3-MeO-PCP. On admission, the blood pressure was 150/104 mm Hg, and the heart rate was 105 beats per minute. Neither hyperthermia nor hypothermia was observed. The SpO_2_ was 96%. Slight psychomotor impairment and dysarthria were noted but the neurological examination was otherwise normal. In particular, there were no consciousness disorders, no limb tremor, and no hypertonia. No biochemical assays were performed for this second intoxication. The patient was discharged to home after 12 hours of clinical monitoring.

## 3 Discussion

### 3.1 Clinical and biochemical manifestations

As with many NPSs, detailed biochemical and clinical data on 3-MeO-PCP intoxication are lacking.^[18]^ Here, we described a case of intoxication with 3-MeO-PCP alone, following oral consumption of 200 mg. The main clinical findings were high blood pressure, tachycardia, neurological manifestations (confusion, hypertonia, nystagmus, and agitation), and rash. Hyperlactatemia (2.6 mmol/L) was the main biochemical finding. In the second intoxication (after the patient had sniffed 50 mg of 3-MeO-PCP), high blood pressure, tachycardia, and neurological manifestations (psychomotor impairment and dysarthria) were present but with less severity. In both instances, the patient was discharged after his clinical status had improved rapidly.

In a review of the literature, we found only 9 other reports of nonfatal intoxications with 3-MeO-PCP alone.[^4^,^10^,^15^] The main clinical and biochemical findings in the 10 cases (ie, including the present report) are summarized in Table 1.

**Table 1:**
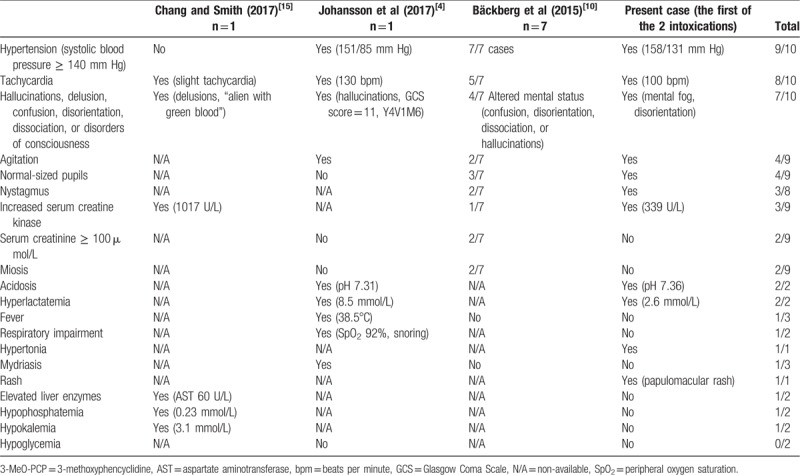
Clinical and biochemical findings in intoxications with 3-MeO-PCP alone.

High blood pressure seems to be a highly sensitive sign of intoxication with 3-MeO-PCP. It was noted in all cases other than that reported by Chang and Smith,^[15]^ although it was suggested that high blood pressure might have disappeared before the initiation of medical care. Indeed, Chang and Smith did not report on the time interval between consumption and medical care. In our patient, the diastolic blood pressure value was particularly high (131 mm Hg). Tachycardia is also a very prevalent sign, and was always (when specified) sinus tachycardia.

Neurological signs are frequent but vary from one case to another. Chang and Smith^[15]^ reported on a patient who was unconscious and then confused. In the case of Johansson et al,^[4]^ the patient was initially unconscious (Glasgow Coma Scale score: 11) and then developed agitation. Both patients had hallucinations. Bäckberg et al^[10]^ observed confusion, disorientation, dissociation, and/or hallucinations in 4 of their patients. Three patients presented with agitation.[^4^,^10^] Our patient had nystagmus, as did 2 patients in the study by Bäckberg et al.^[10]^ Hypertonia was not detected in other intoxications with 3-MeO-PCP alone but was reported in a case of polydrug intoxication involving 3-MeO-PCP and methamphetamine^[5]^; the latter drug alone can trigger muscle twitches.^[19]^ Our patient did not report hallucinations or delusions but he was not specifically questioned about the presence or absence of these symptoms. It is also possible that hallucinations or delusions only appear in patients with a history of psychotic disorders. For example, auditory hallucinations and delusion were reported in a patient who had stopped taking his treatment for schizophrenia.^[15]^

Rash has never been previously mentioned in intoxications with 3-MeO-PCP, either taken alone[^4^,^10^,^15^] or in combination with other substances.[^5^,^9^,^11^] Our patient developed a rash 2 hours after midazolam had been administered. Rash has already been described in this context, although it usually appears sooner after the midazolam injection.[^20^,^21^] Furthermore, we did not observe skin rash during the second intoxication. Hence, a rash-inducing effect of midazolam (and not 3-MeO-PCP) cannot be ruled out.

Our patient's body temperature was normal, as was found in all but 1 of the cases of intoxication with 3-MeO-PCP alone^[4]^: a patient described by Johansson et al^[4]^ had fever (38.5°C) and developed lactic acidosis (pH: 7.31; hyperlactatemia: 8.5 mmol/L). The latter was also the only patient to present respiratory impairments: snoring and a low SpO_2_ (92%), leading to intubation.

A patient from the study by Bäckberg et al^[10]^ presented with rhabdomyolysis. Another case of elevated CK (1017 UI/L) was reported, along with elevated troponin I (0.21 ng/mL); however, an electrocardiogram and echocardiography did not show any significant impairments.^[15]^

With regard to the other abnormal biochemical features, acute kidney failure with a serum creatinine level above 100 μmol/L was reported in 2 of the 7 patients in the study by Bäckberg et al.^[10]^ The severity of these cases and the acute vs chronic nature were not reported. Likewise, it was not reported whether the patient with rhabdomyolysis also had acute kidney failure.

Hypophosphatemia was noted in 2 cases, with values of 0.7 mg/dL (0.22 mmol/L)^[15]^ and 0.55 mmol/L.^[5]^ In the second case, the intoxication also involved methamphetamine. No cases of hypoglycemia were noted. The only abnormal findings for hepatic function were a slightly elevated aspartate aminotransferase level (60 U/L) probably related to rhabdomyolysis, and elevated indirect bilirubinemia (3.7 mg/dL).^[15]^

The clinical picture associated with PCP intoxication (a compound that is better known than its methoxylated analogues) primarily includes nystagmus, retrograde amnesia, hypertension, and agitation.^[22]^ Rhabdomyolysis is a rare but known effect of this intoxication (with an estimated incidence of 2.5%^[23]^) that can be complicated by acute kidney failure. Fever, tachycardia, and high blood pressure are frequent.^[23]^

### 3.2 Dose–effect relationship

The 3-MeO-PCP doses and administration routes are not usually reported in the literature. Here we described two 3-MeO-PCP intoxications in the same patient, with different doses (200 mg, then 50 mg), different administration routes (oral, then nasal), and different levels of severity. The first intoxication required admission to the intensive care unit and 24 hours of monitoring, whereas the second intoxication (with less severe manifestations) required only 12 hours of monitoring in the emergency department.

A few hours after oral consumption (and half an hour after admission), our patient's peripheral blood concentration of 3-MeO-PCP was 71.1 ng/mL. The concentration was not available for the second intoxication, due to the absence of biological samples. The relationship between the blood concentration of 3-MeO-PCP and the clinical effects is similar to that reported in the literature. As in the present case, the 3-MeO-PCP concentrations reported in the literature were mostly below 110 ng/mL for nonfatal intoxications, and from 120 to 380 ng/mL for fatal intoxications.^[10]^ These findings are suggestive of a dose–effect relationship in cases of 3-MeO-PCP intoxication.

### 3.3 Confusion between 3-MeO-PCP and its isomers

In view of the patient's account and the label on the sachet, we first confirmed that the powder contained 3-MeO-PCP. Indeed, we were, thus, able to detect this compound in the patient's blood and urine samples. Several analytical techniques (ie, liquid chromatography with diode array detection, gas chromatography mass spectrometry, and LC-HRMS) were needed to differentiate 3-MeO-PCP from its isomers, such as 2-MeO-PCP and 4-MeO-PCP. At the time when the present case occurred (2018), these MeO-PCPs were not listed as controlled substances in France. Consumption of 3-MeO-PCP was first reported in 2014.

Allard et al^[2]^ described another case of confusion between the doses of 3-MeO-PCP and 4-MeO-PCP. As in the present case, the patient took 200 mg of 3-MeO-PCP, thinking that he was consuming 4-MeO-PCP. This intoxication was more serious, because it resulted in coma and required tracheal intubation for 4 days. However, the intoxication described by Allard et al also involved MDMA, methadone, venlafaxine, oxazepam, and another NPS (AB-FUBINACA). These cases emphasize the risk of dosing errors caused by the similarity between the names “3-MeO-PCP” and “4-MeO-PCP.”

### 3.4 Management

Treatment is symptomatic. No validated antidotes are available. In particular, flumazenil and naloxone have not proved to be effective.^[5]^ A 3-MeO-PCP user reported that *N*-phenylacetyl-L-prolylglycine ethyl ester (Noopept) was effective but this observation has not been confirmed on a larger scale.^[24]^

Some patients required intensive care.[^4^,^10^] In our case, the main treatments were midazolam and intravenous fluids (initially saline solution). It is probable that the choice of saline solution was justified by

1. the presence of mottled skin on the knees, and
2. tachycardia, despite high blood pressure.

3-MeO-PCP's half-life in vivo (estimated at 10 and 11 hours by 2 different research groups)[^4^,^10^] explains the rapid clinical improvement observed in 3-MeO-PCP-only intoxications. Therefore, dialysis and the use of a molecular adsorbent recirculating system are unlikely to be relevant in 3-MeO-PCP intoxications. To the best of our knowledge, these methods have not been tested in this indication. In PCP intoxication, neither hemodialysis nor peritoneal dialysis showed useful results.[^25^,^26^] It was suggested that hemodialysis was ineffective because PCP redistributed from lipid stores to blood after dialysis had ceased.^[26]^

## 4 Conclusion

We presented an instance of the new problem of NPS consumption: a little-known molecule purchased on the Internet that is difficult to identify and whose effects have not been characterized clinically. We described a nonfatal intoxication due to 3-MeO-PCP alone, followed by a second incident in the same patient. By characterizing these incidents and comparing them with the clinical, biochemical, and toxicological data in the literature, we hope to have raised levels of knowledge and facilitated the future management of intoxications with PCP derivatives.

## Author contributions

**Conceptualization:** Jean-Sebastien Allain.

**Supervision:** Jean-Sebastien Allain, Alain Baert, Isabelle Morel, Renaud Bouvet, Thomas Gicquel.

**Validation:** Jean-Sebastien Allain, Renaud Bouvet, Thomas Gicquel.

**Writing – original draft:** Antoine Berar, Thomas Gicquel.

**Writing – review and editing:** Antoine Berar, Sophie Allard, Charles Lefevre, Thomas Gicquel.
